# Gyrotactic cluster formation of bottom-heavy squirmers

**DOI:** 10.1140/epje/s10189-022-00183-5

**Published:** 2022-03-18

**Authors:** Felix Rühle, Arne W. Zantop, Holger Stark

**Affiliations:** Institut für Theoretische Physik, Technische Universität Berlin, Hardenbergstr. 36, D-10623 Berlin, Germany

## Abstract

Squirmers that are bottom-heavy experience a torque that aligns them along the vertical so that they swim upwards. In a suspension of many squirmers, they also interact hydrodynamically via flow fields that are initiated by their swimming motion and by gravity. Swimming under the combined action of flow field vorticity and gravitational torque is called gyrotaxis. Using the method of multi-particle collision dynamics, we perform hydrodynamic simulations of a many-squirmer system floating above the bottom surface. Due to gyrotaxis they exhibit pronounced cluster formation with increasing gravitational torque. The clusters are more volatile at low values but compactify to smaller clusters at larger torques. The mean distance between clusters is mainly controlled by the gravitational torque and not the global density. Furthermore, we observe that neutral squirmers form clusters more easily, whereas pullers require larger gravitational torques due to their additional force-dipole flow fields. We do not observe clustering for pusher squirmers. Adding a rotlet dipole to the squirmer flow field induces swirling clusters. At high gravitational strengths, the hydrodynamic interactions with the no-slip boundary create an additional vertical alignment for neutral squirmers, which also supports cluster formation.

**Supplementary Information:**

The online version contains supplementary material available at 10.1140/epje/s10189-022-00183-5.

## Introduction

The collective dynamics of active entities features intriguing pattern formation on different length scales [1–5]. It also offers the prospect of manufacturing new materials by aggregating synthetic agents [6–8]. Biotic cluster or patch formation of phytoplankton has profound influences on oceanic ecology [9–11], while bacteria forming biofilms endanger human health [12, 13]. Investigating how active particles react on external fields reveals novel aspects of these non-equilibrium systems [14, 15]. This includes sedimentation of active particles [16–21], microswimmers in magnetic fields [22–24], their pearling transition and plume formation in microchannels [25, 26], as well as the question of optimal steering of active entities under flow and in a potential landscape [27–29].

In this article, we focus on microswimmers under gravity, which is of utmost importance for artificial and biological systems alike [16–18, 20, 30–33]. The non-equilibrium sedimentation of active particles has attracted a lot of attention [16–18, 20, 34], revealing an activity-dependent sedimentation length [17], polar order [18], and convection [34]. Under gravity often a gravitational torque acts on microswimmers due to their non-spherical shape or a non-uniform mass distribution, which is responsible for negative gravitaxis [19, 31, 32] and plume formation [30, 35–37].

The collective motion of microswimmers is profoundly influenced by hydrodynamic flow fields, which they create themselves [38–43]. Microswimmers residing at a bottom surface due to strong gravity show hovering and trapped states [21, 44, 45] as well as raft and swarm formation [46, 47], due to hydrodynamic wall interactions. Also external forces induce fluid flow, for example, when passive colloids sediment under gravity [48]. An example from active matter are microswimmers in a harmonic trap. They experience novel pattern formation by creating a fluid pump [49, 50].

Gyrotaxis refers to directed locomotion resulting from a combination of gravitational and viscous torques in a flow [51–53]. In particular, it applies to the action of fluid vorticities on microswimmers [51, 53]. As such, it is responsible for the dancing motion of Volvox algae [54], focussing in channel flow [55], the formation of layers [10] as well as patches of phytoplankton [56], and convective patterns in algae and bacteria [30, 57–59]. These examples show the importance of gyrotaxis for biological systems. They motivated us, in an earlier article, to perform hydrodynamic simulations using the method of multi-particle collision dynamics in order to systematically study the dynamics and gyrotaxis of bottom-heavy squirmers. The latter are generic model microswimmers [60]. Depending on the strength of gravity and bottom heaviness, we identified different dynamical states including inverted sedimentation, plumes and convective rolls, as well as spawning clusters.

**Table 1:** Overview of physical parameters

Parameter	Meaning
*R*	Squirmer radius
$v_0$	Active velocity
$v_\mathrm {sed}$	Sedimentation velocity
$\alpha$	Velocity ratio
$\beta$	Force dipole parameter
$\chi$	Rotlet dipole parameter
$r_0$	Center of mass shift
$r_0/(R\alpha )$	Rescaled bottom-heavy torque

**Table 2:** Overview of simulation parameters

Parameter	Meaning
$a_0$	Edge length of collision cell
$m_0$	Mass of fluid particle
$T_0$	Temperature of fluid
$\varDelta t$	Duration of streaming step
$n_\mathrm {fl}$	Fluid particle number density
$\eta$	Dynamic fluid viscosity

In this article, we continue our work on gyrotaxis of bottom-heavy squirmers but now concentrate on moderate densities and strong gravity such that they float above but close to the bottom surface. Only with increasing gravitational torque gyrotactic cluster formation sets in as a subtle balance between hydrodynamic and gravitational torques. We thoroughly characterize the cluster formation of neutral squirmers for different areal densities and torques using different quantities such as the vertical density profile, mean cluster size, and radial distribution function. We also discuss the influence of squirmer flow fields from pusher and puller squirmers. While pusher squirmers do not exhibit noticeable cluster formation, larger torques are needed to observe it for puller squirmers. Furthermore, a hydrodynamic rotlet dipole realized, for example, by bacteria also weakens cluster formation.

The article is structured as follows. In Sect. 2 we present the squirmer model and describe in detail the hydrodynamics of this model swimmer under gravity. Furthermore, we introduce the method of multi-particle collision dynamics to simulate the flow field generated by the squirmers. In Sect. 3 we present our simulation results and we conclude in Sect. 4.

## Methods

We simulate squirmer microswimmers under gravity using the mesoscale simulation technique of multi-particle collision dynamics. The squirmers move in a three-dimensional simulation box with no-slip walls at the bottom and at the top and periodic boundary conditions in lateral direction. Tables 1 and 2 summarize our physical and simulation parameters, respectively.

### Squirmer model

We use the squirmer model introduced by Lighthill and Blake [61, 62] in order to investigate gyrotactic cluster formation of microswimmers. The original idea of the model is to consider a spherical particle with a surface velocity field and expand it into appropriate base function to model the surface actuation of a ciliated microswimmer. A more general ansatz includes chiral surface flow and was presented in Ref. [63], which we follow here. We work with an axisymmetric tangential flow field.

Then, the surface slip velocity for a squirmer with radius *R* that swims along the unit vector ${\mathbf {e}}$ can be expressed as

1 $$\begin{aligned} {\mathbf {u}}_\mathrm {sq}({\mathbf {r}})\vert _{r=R}= & {} \sum _{n=1}^{\infty }B_n \frac{2 P^\prime _n({\mathbf {e}}\cdot \mathbf {\hat{r}})}{n(n+1)}\left[ - {\mathbf {e}} + ({\mathbf {e}}\cdot \mathbf {\hat{r}}) \mathbf {\hat{r}} \right] \nonumber \\&+ \sum _{n=1}^{\infty }C_n P^\prime _n({\mathbf {e}}\cdot \mathbf {\hat{r}})\left[ {\mathbf {e}} \times \mathbf {\hat{r}}\right] . \end{aligned}$$

Here $P^\prime _n$ refers to the first derivative of the *n*th Legendre polynomial $P_n$.

Often, one truncates this expansion after the second order. The mode $B_1$ is necessary for self-propulsion where $v_0 = \frac{2}{3}B_1$ is the swimming velocity. Additionally, we consider the modes $B_2$ and $C_2$, which we express in terms of the squirmer parameter $\beta = B_2/B_1$ and chirality parameter $\chi = C_2/B_1$, see for example Ref. [64]. The parameter $\beta$ switches between neutral squirmers ($\beta =0$), pullers ($\beta >0$) and pushers ($\beta < 0$).

#### Hydrodynamics of bottom-heavy squirmers

We collect all the flow and vorticity fields of a bottom-heavy squirmer and thereby provide an overview on the hydrodynamics of our squirmer system. While we use periodic boundary conditions in our numerical scheme, we do not include them in the following presentation.

*Flow and vorticity fields* The flow field of the freely swimming squirmer resulting from the truncated surface field in eq. (1) is [63]

2 $$\begin{aligned} {\mathbf {u}}_\mathrm {sq}({\mathbf {r}})= & {} \frac{3}{2}v_0 \left[ \frac{1}{3}\frac{R^3}{r^3}\left( -\mathbf {{\mathbf {e}} + 3({\mathbf {e}}\cdot \mathbf {\hat{r}})\mathbf {\hat{r}}}\right) \right. \nonumber \\&- \frac{\beta }{2}\frac{R^2}{r^2}\left( -1+ 3({\mathbf {e}}\cdot \mathbf {\hat{r}})^2\right) \mathbf {\hat{r}} \nonumber \\&+ \left. 3\chi \frac{R^3}{r^3} ({\mathbf {e}}\cdot \mathbf {\hat{r}}) {\mathbf {e}} \times \mathbf {\hat{r}} + {\mathcal {O}}\left( \frac{R^4}{r^4}\right) \right] , \end{aligned}$$

where we have written the different contributions such that one immediately recognizes the source dipole (first term), force dipole (term with $\beta$) and rotlet dipole (term with $\chi$).

Equation (2) shows that a squirmer always creates a source dipole field, which becomes the leading order for the neutral squirmer ($\sim r^{-3}$). For a pusher or puller squirmer, a force dipole field is added ($\sim r^{-2}$), whereas $\chi \ne 0$ refers to the rotlet dipole. Pushers model propulsion originating from the back of a swimmer’s body, such as rotating flagella, whereas pullers are a more suitable description for a breast-stroke type of motion. Pusher and puller fields are the most typical hydrodynamic modes considered in microswimmer systems [60, 65]. Furthermore, the rotlet dipole field is an important aspect of many swimming organisms, for example, of bacteria with counter-rotating flagella and cell body. It has been studied in some previous works [64, 66].

The complete hydrodynamic flow field generated and experienced by a collection of squirmers is strongly determined by their manifold hydrodynamic interactions and is therefore analytically untractable. However, we can still gain insights from the single squirmer fields and their different contributions, which can be ordered according to their radial decay. This can help to interpret numerical results of their collective dynamics.

In our case, the self-created flow fields of the squirmers from Eq. (2) are not the only contributions. Also their gravitational force and bottom-heaviness generate flow fields. Furthermore, the no-slip boundary condition on the bottom surface affects the hydrodynamics by creating a wall-induced flow [67]. Fortunately, the Stokes equations are linear, which allows us to superimpose all occuring fields.

*Contributions from gravity* The first part comes from the gravitational force acting on the squirmer and is the same as the flow field of a passive sphere dragged through the fluid. Therefore, a squirmer under gravity always induces the flow fields of a stokeslet and a source dipole [68]. The latter adds to the source dipole of the squirmer [63]. Additionally, since the center of mass of the squirmer is shifted by a distance $r_0$ from its geometric center, it experiences bottom heaviness, which aligns the swimming direction $\mathrm {e}$ along the vertical ${\mathbf {e}}_z$. Due to the gravitational torque ${\mathbf {T}}_\mathrm {bh} = mgr_0 {\mathbf {e}} \times {\mathbf {e}}_z$ acting on the squirmer, it rotates with angular velocity $\mathbf {\Omega }_\mathrm {bh} = {\mathbf {T}}_\mathrm {bh} / (8\pi \eta R^3)$ and thereby induces a rotlet field. The velocity fields of both contributions are [60, 63, 67]:

3 $$\begin{aligned} {\mathbf {u}}_\mathrm {grav}({\mathbf {r}})= & {} \frac{1}{4}v_\mathrm {sed} \left[ -3 \frac{R}{r}\left( {\mathbf {e}}_z + \frac{z}{r}\mathbf {\hat{r}}\right) \nonumber \right. \\&\left. + \frac{R^3}{r^3}\left( -{\mathbf {e}}_z + 3\frac{z}{r}\mathbf {\hat{r}}\right) \right] \end{aligned}$$

4 $$\begin{aligned} {\mathbf {u}}_\mathrm {bh}({\mathbf {r}})= & {} \frac{3}{4}v_0 \frac{r_0}{R \alpha }\frac{R^2}{r ^2}\left( ({\mathbf {e}}\cdot \hat{{\mathbf {r}}}){\mathbf {e}}_z - \frac{z}{r}{\mathbf {e}}\right) \,. \end{aligned}$$

Here, we introduced the dimensionless parameters

$\alpha = v_0 / v_\mathrm {sed}$ and $r_0/ (R\alpha )$, with the bulk sedimentation velocity $v_\mathrm {sed}=mg/(6\pi \eta R)$ and the center-of-mass shift $r_0$. The parameter $\alpha$ measures the swimming velocity relative to the sedimentation velocity and $r_0 / (R \alpha )$ is a unitless strength of the gravitational torque.

In these units the related angular velocity becomes [19]:

5 $$\begin{aligned} \mathbf {\Omega }_\mathrm {bh} = \frac{3}{4}\frac{v_0}{R}\frac{r_0}{R\alpha } {\mathbf {e}}\times {\mathbf {e}}_z \, . \end{aligned}$$

The actual flow field of the squirmer together with the gravity-induced contributions constitute the hydrodynamic signature of our problem and give the relevant flow fields in leading order in 1/*r*.

Importantly, the non-zero vorticities of these flow fields reorient nearby squirmers or the squirmer itself when it interacts hydrodynamically with a wall. Since the vorticity of a source dipole is zero, we only need to consider the vorticity from the gravity-induced stokeslet and rotlet as well as the vorticity from the force dipole of squirmers with $\beta \ne 0$. Using the definition of the vorticity, $\varvec{\omega }({\mathbf {r}}) = \nabla \times {\mathbf {u}}({\mathbf {r}})$, the respective vorticities of the stokeslet, rotlet, and force dipole of a bottom-heavy squirmer are

6 $$\begin{aligned} \varvec{\omega }_{S}= & {} \frac{3}{2}v_\mathrm {sed}\frac{R}{r^2}\mathbf {\hat{r}}\times {\mathbf {e}}_z, \end{aligned}$$

7 $$\begin{aligned} \varvec{\omega }_{R}= & {} \frac{3}{4}v_\mathrm {0}\frac{r_0}{R\alpha }\frac{R^2}{r^3}\nonumber \\&\left( 2{\mathbf {e}}\times {\mathbf {e}}_z - 3 \left( ({\mathbf {e}}\cdot \mathbf {\hat{r}})\mathbf {\hat{r}}\times {\mathbf {e}}_z - \frac{z}{r}\mathbf {\hat{r}}\times {\mathbf {e}}\right) \right) , \end{aligned}$$

8 $$\begin{aligned} \varvec{\omega }_{Fd}= & {} -\frac{9}{2}v_0\beta \frac{R^2}{r^3}({\mathbf {e}}\cdot \mathbf {\hat{r}})\left( {\mathbf {e}}\times \mathbf {\hat{r}}\right) . \end{aligned}$$

Note that the vorticity of the rotlet, $\varvec{\omega }_{R}$ from eq. (7), vanishes for ${\mathbf {e}}\rightarrow {\mathbf {e}}_z$ and therefore this contribution is typically small.

Now, placing a second squirmer at position ${\mathbf {r}}_2$ in the flow field of a sufficiently distant first squirmer, its angular and translational velocities are determined by the collected far field expressions. Using Faxén’s theorem [68, 69], the angular velocity up to terms proportional to $1/r_2^3$ is given by $\frac{1}{2}\left( \varvec{\omega }_{S}({\mathbf {r}}_2) + \varvec{\omega }_R({\mathbf {r}}_2)\!+\! \varvec{\omega }_{Fd}({\mathbf {r}}_2) \right.$ $\left. + {\mathcal {O}}(r_2^{-4})\right)$ and the translational velocity becomes ${\mathbf {u}}_\mathrm {sq}({\mathbf {r}}_2) + {\mathbf {u}}_\mathrm {grav}({\mathbf {r}}_2)$ $+ (R^2/6)$ $\nabla ^2 {\mathbf {u}}_\mathrm {grav}({\mathbf {r}}_2) + {\mathbf {u}}_\mathrm {bh}({\mathbf {r}}_2) + {\mathcal {O}}(r_2^{-4})$, where ${\mathbf {r}}_2$ is the distance vector from the first squirmer to the second.

The collective dynamics in gyrotactic systems is

determined by the competition and balancing of external torque and flow vorticity acting on the microswimmers. One can use this effect to focus swimmers in an external Poiseuille flow [55]. More recently, it has been shown that a prescribed periodic flow can lead to vertical trapping of gyrotactic motile cells [70]. In our case, however, the flow vorticity arises from the flow fields of the neighboring squirmers. Assuming two squirmers on the same height with distance *r*, they rotate each other away from the vertical by an angle $\vartheta$ due to the stokeslet vorticity of eq. (6), in leading order. Balancing this rotation by the angular velocity of eq. (5) due to the gravitational torque gives

9 $$\begin{aligned} \frac{3}{4}\frac{v_0}{R}\frac{r_0}{R\alpha }\sin \vartheta = \frac{3}{4}\frac{v_0}{\alpha }\frac{R}{r^2} \, . \end{aligned}$$

In the following we will identify stable clusters of squirmers in our simulations only when the gravitational torque is sufficiently large. We present a simple argument for this behavior. At the closest possible distance $r=2R$, the angle where both angular velocities in eq. (9) balance each other becomes

10 $$\begin{aligned} \sin \vartheta = \frac{1/(4\alpha )}{r_0/(R\alpha )} \, . \end{aligned}$$

Since $\sin \vartheta \le 1$, such a stable balance requires the lower bound

11 $$\begin{aligned} r_0/(R\alpha ) \ge (4\alpha )^{-1} \end{aligned}$$

for the dimensionless torque. As we will show in Sect. 3.2.1 this gives a good estimate for the torque, where the clusters start to become compact.

*Wall terms* Close to a no-slip wall mirror multipoles have to be added to the hydrodynamic multipoles introduced above, in order to fulfill the no-slip boundary condition [67]. The leading far-field contribution of a squirmer under gravity is the stokeslet, which close to a no-slip wall turns into the Blake tensor [71, 72]; the influence of its mirror multipoles decays with the inverse height. Furthermore, the squirming modes controlled by the parameters $\beta$ and $\chi$ further affect the wall-induced flow field, which depends on the squirmer orientation. For example, vertically oriented pushers are repelled and pullers are attracted to the wall [21].

We are mainly interested in the vorticities of the wall-induced flow fields since they are directly related to gyrotaxis. We give the most relevant terms here following Refs. [21, 67]. By symmetry the mirror image in the Blake tensor does not contribute to the wall-induced vorticity acting on a single squirmer. Therefore, the leading-order contribution for pushers and pullers is the reflected force dipole field, whereas for $\beta = 0$ and $\chi \ne 0$ it is the reflected rotlet-dipole field. Second, a force quadrupole arises as a wall-induced mirror term in the three squirmer multipoles with strengths $B_1$, $B_2$ and $C_2$. In particular, this force quadrupole results from the reflected vorticity field of the source dipole [67] and, therefore, determines the orientation of the neutral squirmer in wall proximity [21]. We write the vorticity or angular velocity ${\varvec{\varOmega }}^{\mathrm {wall}}$ as a function of the distance to the no-slip wall, $\varDelta z$, and the angle $\vartheta$ of the squirmer orientation ${\mathbf {e}}$ with respect to the vertical [67]. Using cylindrical coordinates, we arrive at the following components

12 $$\begin{aligned} \varOmega ^\mathrm {wall}_{\rho }= & {} \frac{81}{32} \frac{v_0}{R} \chi \sin \vartheta \cos \vartheta \frac{R^4}{\varDelta z^4} \end{aligned}$$

13 $$\begin{aligned} \varOmega ^\mathrm {wall}_{\phi }= & {} -\frac{3}{16} \frac{v_0}{R} \sin \vartheta \left( \frac{R^4}{\varDelta z^4} + \frac{3}{2}\beta \cos \vartheta \frac{R^3}{\varDelta z^3}\right) \end{aligned}$$

14 $$\begin{aligned} \varOmega ^\mathrm {wall}_{z}= & {} -\frac{27}{64} \frac{v_0}{R} \chi (1-3\cos ^2\vartheta )\frac{R^4}{\varDelta z^4} \, . \end{aligned}$$

For example, this angular velocity becomes zero for a vertically oriented neutral squirmer with $\beta ,\chi =0$. The additional force-dipole flow field of pushers and pullers changes this stable orientation [21]. Furthermore, it is known that the rotlet dipole induces the wall component $\varOmega ^\mathrm {wall}_{z}$ of eq. (14) that is responsible for circular swimming [66, 73].

### Hydrodynamic simulations

#### Multi-particle collision dynamics

Various successful strategies exist for treating the low-Reynolds-number fluid dynamics of suspended microswimmers. This includes direct discrete simulations, using finite-volume [74] or smoothed-profile [64] methods, the boundary-element method, where an integral equation is solved  [66, 75], or mesoscale techniques such as lattice-Boltzmann simulations [76, 77]. In this article, we use the mesoscopic simulation technique of multi-particle collision dynamics [78–80], which is well established for hydrodynamic simulations of microswimmers, in particular, squirmers [34, 41, 42, 81–85]. In particular, this technique has successfully reproduced the correct squirmer flow fields [81, 86] as well as their near- and far-field interactions [83, 87]. It simulates hydrodynamic flow fields as solutions of the Navier-Stokes equations and also includes thermal noise  [88, 89]. The fluid consists of coarse-grained but point-like fluid particles of mass $m_0$. The algorithm of multi-particle collision dynamics applies a sequence of alternating streaming and collision steps to these particles [80].

During the streaming step with duration $\varDelta t$, the fluid particles move ballistically. During this step one also implements the no-slip boundary condition at bounding walls using the bounce-back rule [90]. It is also used to prescribe the tangential velocity field of Eq. (1) on the squirmer surface, which enables self-propulsion. Furthermore, the suspended squirmers are treated as rigid bodies and their positions and orientations are updated using velocity-Verlet integration [91], which accounts for the gravitational forces and torques acting on them. In order to model the steric repulsion between squirmers and between squirmers and bounding walls, we use a steep WCA potential. We always choose the Reynolds number as small as computationally feasible and arrive at $\mathrm {Re}=0.17$ [60]. This is still a good approximation of the Stokes flow regime. Thus, the acceleration of the swimmer due to the external force is balanced by a friction force, consistent with force-free swimming. For the collision step we introduce a length scale $a_0$ to indicate the range of fluid-particle collisions, which take place in cubic cells of side length $a_0$. On average, we place $n_\mathrm {fl}$ fluid particles into the cells. Both values of $\varDelta t$, as well as $n_\mathrm {fl}$, determine the fluid properties such as viscosity. Further simulation details are described more exhaustively in our previous works [42, 60, 91].

The collisions of the fluid particles inside a collision cell are governed by a specific collision operator or collision rule, which redistributes the relative momentum between the particles keeping the total momentum fixed. Several different collision rules exist for multi-particle collision dynamics [80]. We use the MPC-AT+a algorithm which assures Galilean invariance and angular momentum conservation [80, 92]. It is based on the Andersen thermostat [80, 89], which keeps the thermal energy of the fluid at the fixed value $k_B T_0$. If a collision cell overlaps with a suspended squirmer or a bounding wall, this volume is filled with virtual particles [91, 93] to ensure that the mean density in the collision step is always $n_\mathrm {fl}$. This approach also improves the accuracy of the flow fields and also prevents depletion forces [94]. All in all, the cell length $a_0$, the mass $m_0$ of a fluid particle, and the thermal energy $k_BT_0$ form the units of length, mass, and energy. From this we can derive other units, in particular, for time $a_0\sqrt{m_0/k_BT_0}$ and velocity $\sqrt{k_BT_0/m_0}$.

#### Implementation and parameters

We simulate the MPCD fluid inside a rectangular system with height *H* and a quadratic cross section with edge length *L*. We use periodic boundary conditions in lateral direction and two no-slip walls in vertical direction. Usually, we have $H=L=108a_0$, *i.e.*, a cubic simulation box. For the fluid we use the duration of the streaming step $\varDelta t = 0.02 a_0\sqrt{m_0/k_BT_0}$ and the fluid-particle number density $n_\mathrm {fl}=10$. This choice of parameters results in a viscosity of $\eta =16.05\sqrt{m_0k_BT_0}/a_0^2$ [95]. Our squirmers have $B_1=0.1\sqrt{k_BT_0/m_0}$ and, unless stated otherwise, $\beta =\chi =0$. The resulting swimming velocity is $v_0=0.067\sqrt{k_BT_0/m_0}$, which corresponds to an active Péclet number $\mathrm {Pe}=\frac{Rv_0}{D_T} = 330$, comparable, for example, to bacteria [37, 96]. Furthermore, the squirmers have a radius $R=4a_0$, and the relevant ballistic time scale $v_0/R$ amounts to around $3000\varDelta t$ [60]. To set the parameter $\alpha$, we choose a moderate strength of gravity that always creates a sedimentation velocity larger than $v_0$, typically $\alpha =0.8$. This prevents squirmers from escaping from the bottom of the system to the top wall.

Due to the high computational cost of hydrodynamic simulations, we compile software for running on multiple processing units simultaneously, *i.e.*, the program is significantly parallelized. Earlier simulations were performed on a high-performance computing cluster using approximately 150 CPU cores. For later simulations we switched to a CUDA implementation, which we ran on graphics processor units.

*Initial condition* We tested two different initial conditions. Either, the squirmers were distributed randomly in the simulation box or we initialized them on a regular grid in the midplane $z=H/2$, all pointing upwards with $\cos \vartheta =1$. This had little effect on the clustering behaviour that we focus on in the following. The only difference we observed was that for the random initialization more squirmers escaped to the top wall for strong bottom-heaviness. Otherwise, these squirmers have no influence on the rest of the simulation, as they stay at the top plate without interfering during the rest of the simulation. Choosing random orientations at the beginning of the simulation or initializing the squirmers in another plane with constant *z* also brought no qualitative change.

## Results

We present our results on gyrotactic clusters and the associated parameter studies in the following. The decisive parameters are the velocity ratio $\alpha$ and the torque measure $r_0/R\alpha$ that we introduced above. In previous studies [34, 60] we investigated large-scale convection and plumes that move over the entire system height. In contrast, we here look at the cluster formation of bottom-heavy squirmers that are constrained to the bottom wall due to strong gravity ($\alpha <1$). However, the squirmers are still capable of moving vertically, in contrast to monolayer formation for $\alpha$ below 0.1 [47]. Thus, we observe the emergence of dynamic clusters from a squirmer suspension that float above the bottom wall.

### Phenomenology of gyrotactic clusters

At the outset we discuss the basic properties of the emergent structures that form under gravity and bottom-heaviness. We present snapshots of the squirmer system and discuss the vertical density profiles and dynamics of the clusters. However, we start by illustrating the coupled dynamics of two squirmers.

#### Gyrotaxis of a squirmer pair

**Fig. 1:**
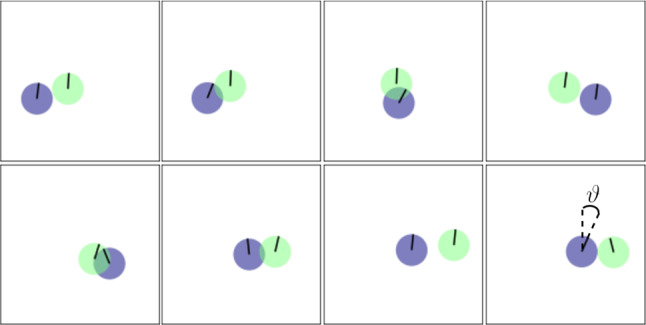
Snapshots of a pair of squirmers performing coupled oscillations above the bottom wall. The simulations used the parameters $\alpha =0.8$ and $r_0/R\alpha =0.16$

Gyrotaxis is often discussed in the context of collective dynamics of microswimmers, but already a two-particle system can illustrate the combined effect of a gravitational torque and fluid flow. For example, Drescher *et al.* calculated the coupled trajectories of two microswimmers based on eqs. (3), (5), and (6), just by considering the stokeslet flow fields and the gravitational torque, and thereby reached good agreement with their experimental data [54]. They found that the dynamical system of two hydrodynamically interacting microswimmers contains oscillatory trajectories when reorientation due to the neighbor’s stokeslet vorticity and due to the own gravitational torque balance each other. In the following, we reproduce such oscillations in our squirmer simulations. We show the simulated oscillations for a pair of squirmers in Fig. 1 and outline the mechanism in the following.

The simulations are performed for $\alpha =0.8$, where single neutral squirmers float above the bottom wall at a height of several squirmer radii *R* [21]. Due to bottom-heaviness, they experience a gravitational torque proportional to $-\sin \vartheta$, where $\vartheta$ is the polar angle between squirmer orientation and vertical (see last snapshot in the bottom row of Fig. 1). Thus, squirmers in bulk tend towards the vertical where the gravitational torque is zero. Two squirmers with such a vertical orientation are shown in the first snapshot in Fig. 1. The neighbor can tilt the orientation vector towards itself due to the vorticity of the stokeslet, as shown in the second snapshot. Balancing reorientations due to vorticity and torque, the squirmers are tilted and thus move towards each other. They eventually pass each other (snapshots 3 and 4), whereupon the sense of the vorticity reverses and the motion repeats itself in the other direction, which is shown in snapshots 5-8. The external torque is important to stabilize the pair so that the distance of the squirmers does not increase too much after they have passed each other, which would otherwise destroy the oscillations [54]. During a passing trajectory of a squirmer, it sinks to a lower height due its tilted orientation and swims around and slightly underneath the other squirmer. The squirmers do not penetrate each other and the overlap seen in the snapshots is merely an effect of the projection. Interestingly, even for large torques the squirmer orientations are not completely frozen, but pair formation still occurs with a smaller oscillation amplitude. Since the gravitational torque approaches zero for a vertical orientation, a small tilt and thereby horizontal motion can still be achieved by the vorticity in the flow field.

#### Formation of clusters

Now, we give an overview over what happens to the oscillatory motion in systems with a larger number of neutral squirmers.

In Fig. 2 we show

snapshots of squirmer configurations seen from the top for different torque strengths and squirmer numbers. We used a constant $\alpha =0.8$. The third row with $r_0/R\alpha =0.32$ is available in the supplement as videos M1-M3. These videos also show a side view of the system.

**Fig. 2:**
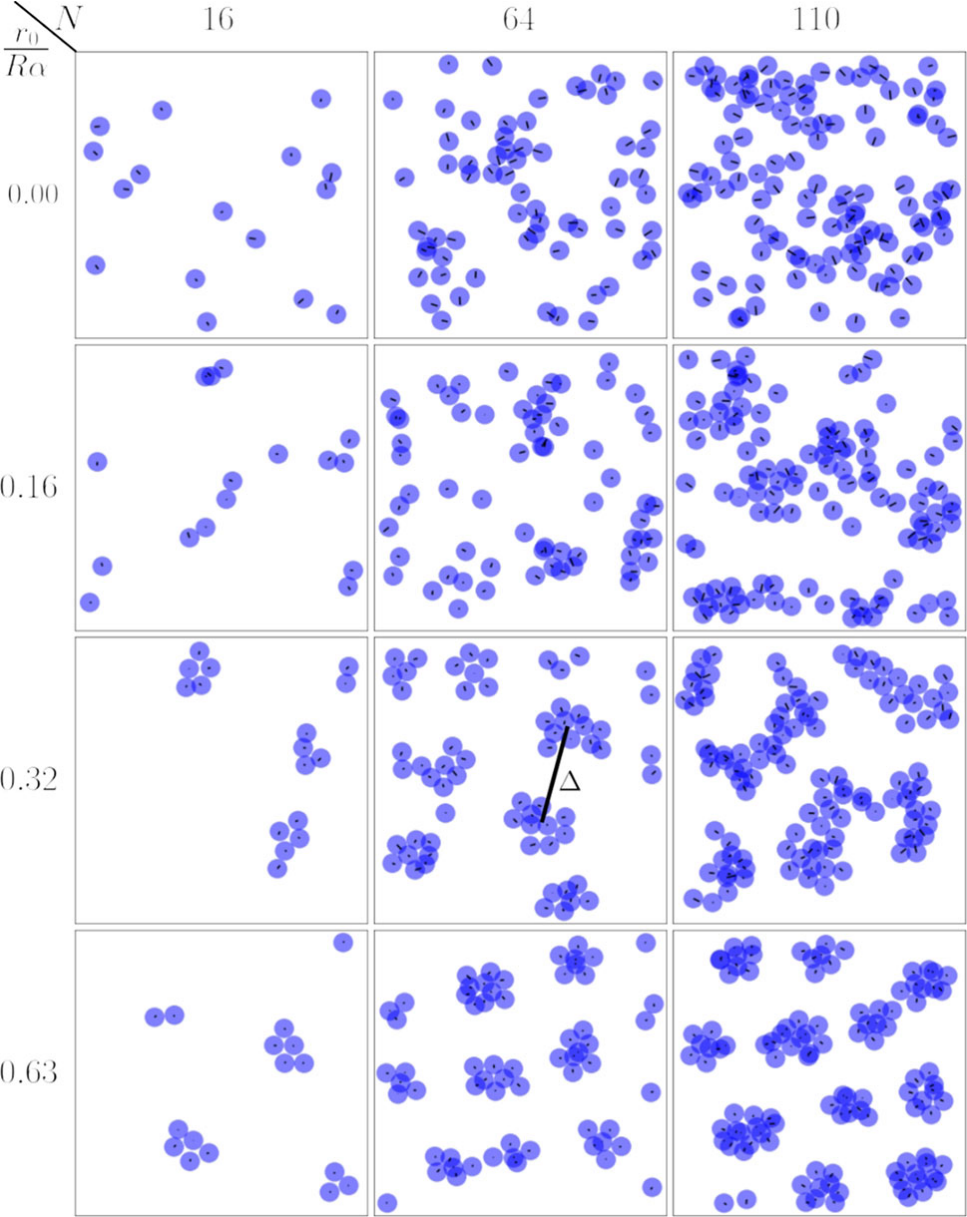
Top view of snapshots of squirmer configurations. They show clusters that form in simulations with different numbers of squirmers *N* and torque strengths $r_0 / (R\alpha )$. Other parameters are $\beta = 0$ and $\alpha =0.8$

The snapshots show that squirmer clusters emerge for different squirmer numbers and torque values and spread over the whole plane with a typical cluster distance $\varDelta$ as indicated in the central snapshot for $r_0/R\alpha =0.32$. These clusters only emerge at finite gravitational torques. At zero torque (top row), squirmers briefly touch during collisions but do not form clusters. Video M4 shows this case for $N=64$. Without the gravitational torque and the strong tendency to align with the vertical, only hydrodynamic interactions and thermal noise determine the orientations of squirmers. They tilt more strongly and, therefore, exhibit stronger horizontal motion. As explained above, squirmers swim past each other and cannot form stable structures.

**Fig. 3:**
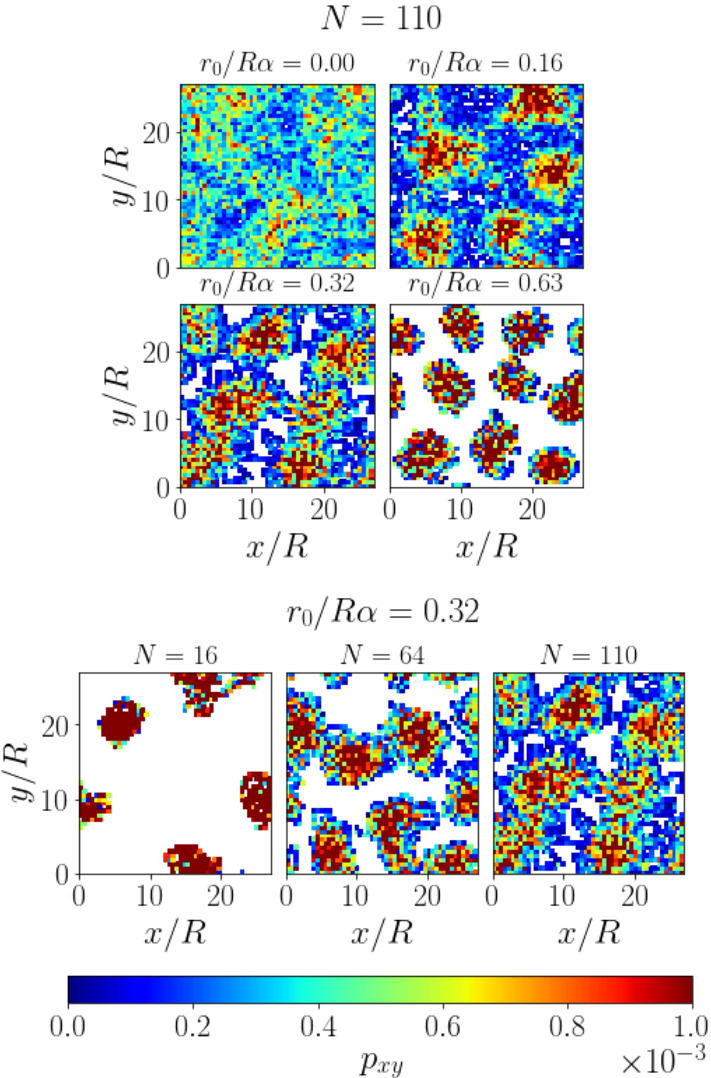
Density profiles after averaging over $10^5$ time steps. Top: for different torque values and $N=110$ squirmers. Bottom: for different *N* and torque value $r_0/R\alpha =0.32$

In Fig. 3 we show the density profiles of the squirmer system in the horizontal plane. In the top panel the torque increases from top left to bottom right while $N=110$.

The density profiles were obtained by averaging over $10^5$ time steps and over all squirmers in the bottom half of the system. At zero torque the density profile is unstructured and almost uniform. There is some patchiness which we attribute to the aligning effect of the bottom wall, leading to floating squirmers that stay in the same spot for longer times [21].

At $r_0/R\alpha =0.16$ we observe that squirmers form clusters which are stable during a longer time

(Fig. 3, top).

There is still considerable horizontal motion and squirmers frequently join and leave the clusters,

therefore the space around the density peaks is still well explored. With increasing torque (*e.g.*, last two rows in Fig. 2) the clusters become more static and compact. This is understandable since a stronger vertical alignment obstructs the escape of squirmers from clusters because the horizontal velocities are smaller. According to the two-dimensional density profile for $r_0/R\alpha =0.32$ in Fig. 3, top, the squirmers explore less space in the horizontal plane and the clusters indeed become more compact, which is most clearly seen for $r_0/R\alpha =0.63$. The same effect occurs for the oscillating squirmer pairs, *i.e.*, the horizontal extent of the clusters decreases with increasing torque. In parallel, the total number of clusters increases as the right-most column of Fig. 2 shows for $N=110$.

The bottom plot of Fig 3 shows how the clustering of squirmers evolves when their number increases at constant torque. An increasing *N* can also be expressed as an increasing area fraction $N\pi R^2/L^2$, where *L* is the edge length of the simulation box. We observe a stronger horizontal motion, which is visible from the disappearance of unvisited space and less pronounced peaks in density. This makes sense since each additional squirmer introduces a new stokeslet, which reorients neighbors away from the vertical.

**Cluster dynamics** Drescher *et al.* coined the term “minuet dance” for the oscillating two-squirmer motion [54]. For larger clusters the motion

looks less regular and consists of squirmers switching their positions inside the cluster as videos M1-M3 show. We stress again that the clusters become more static with increasing acting torque. The cluster reorganization sometimes creates symmetrical structures, such as pentagons or hexagons, as video M2 illustrates. However, these configurations do not persist. A similar effect was reported in experiments with active emulsion droplets [97]. They form clusters that also rearrange frequently at high Péclet number.

#### Vertical density profile

**Fig. 4:**
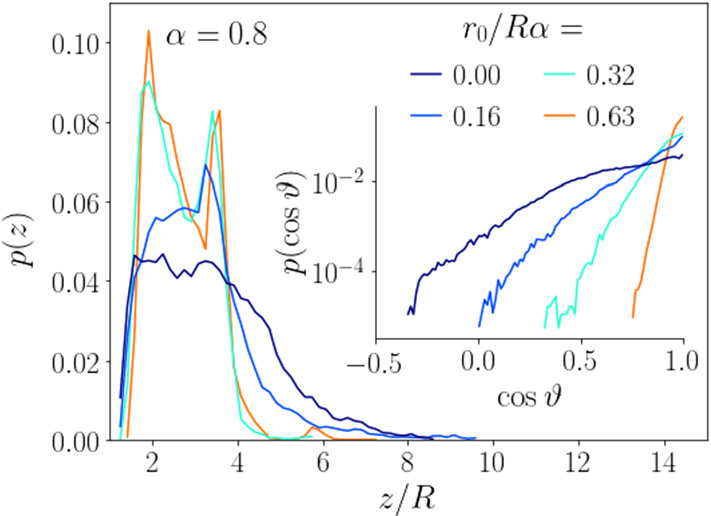
Vertical density profile plotted versus squirmer height *z*/*R* for different torque values at $\alpha = 0.8$ and for $N=64$ squirmers. Inset: Distribution functions of vertical orientation angle $\theta$

**Stacking** The top-view snapshots in Fig. 2 show occasional stacking of squirmers, visible as overlaps, especially at the higher squirmer number in the right column. We stress that these overlaps are due to the projection of the squirmers on one plane. They only occur when the squirmers move on different heights and, of course, the squirmers do not penetrate each other. In order to investigate the vertical structure more closely, we show the vertical density profiles in Fig. 4 for different torque values. The stacking can clearly be seen as a double peak at the two higher torque values. More precisely, a bilayer is formed since the distance between the two peaks in Fig. 4 for $r_0/R\alpha =0.32$ and 0.63 is approximately one squirmer diameter.

**Collective sinking** Note that the vertical density profile at zero rescaled torque reaches to larger heights compared to the non-zero values. Thus, the squirmers are more often found at larger heights. This is the case, even though the orientational distribution gets more strongly peaked at the vertical orientation ($\cos \theta = 1$) with increasing $r_0/R\alpha$ (see inset of Fig. 4). The reason for this counter-intuitive behaviour is a reduced translational friction coefficient per squirmer when they form clusters [48, 98]. At zero torque clusters do not exist and squirmers sink individually due to gravity. In contrast, for higher torques they sediment faster within clusters since hydrodynamic friction is reduced due to hydrodynamic interactions, as was studied in detail in Ref. [60]. This leads to stronger effective sinking, even though the alignment is more vertical.

### Parameter study

We present a more detailed parameter study of squirmer clustering due to gyrotaxis. We characterize clusters by their radius and mean particle number, look at the radial distribution function, study the influence of the squirmer flow field beyond the neutral squirmer, and look closer at the influence of gravity by varying $\alpha$.

#### Cluster radius and size

**Fig. 5:**
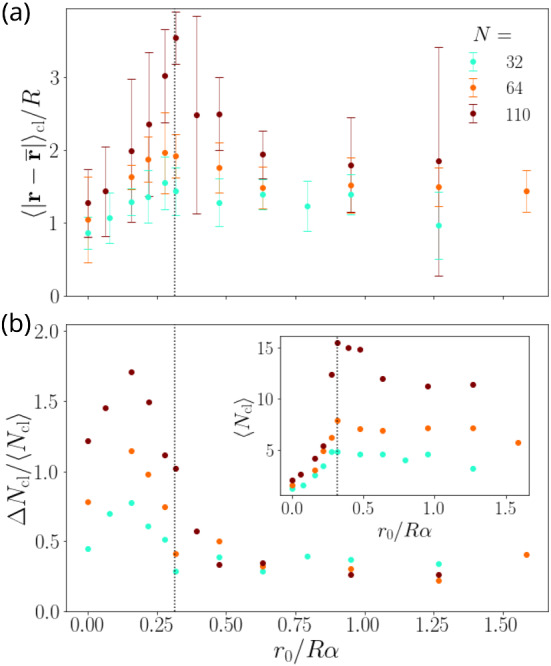
(a) Mean cluster radius $\langle \vert {\mathbf {r}}-\mathbf {\overline{r}}\vert \rangle _\mathrm {cl}$ in units of *R* plotted versus torque value $r_0/R\alpha$ for different squirmer numbers *N*. (b) Normalized standard deviation $\varDelta N_\mathrm {cl}/\langle N_\mathrm {cl} \rangle$ and inset: mean number of squirmers in a cluster $\langle N_\mathrm {cl} \rangle$. The dotted vertical lines show the equality condition of eq. (11) for $\alpha =0.8$

In order to demonstrate how the gravitational torque influences the sizes of the emerging clusters, we define measures for the cluster extension and size. We plot them versus the torque value in Fig. 5 for different squirmer numbers *N*. First, to quantify the cluster extension, we introduce a mean cluster radius by determining the average distance of a squirmer to the cluster center at $\mathbf {\overline{r}}$, $\langle \vert {\mathbf {r}}-\mathbf {\overline{r}}\vert \rangle _\mathrm {cl}$. Here, besides the time average, $\langle \ldots \rangle _\mathrm {cl}$ also means averaging over all squirmers in a cluster and then over all clusters. Second, for the cluster size we calculate the mean number of squirmers in a cluster, $\langle N_\mathrm {cl} \rangle$. We also consider the standard deviation $\varDelta N_\mathrm {cl} = [\langle (N_\mathrm {cl} - \langle N_\mathrm {cl} \rangle )^2 \rangle ]^{1/2}$ to have a measure how much the cluster size varies between the different clusters and also in time. To determine all these quantities, we define a cluster as a set of squirmers, where for each squirmer the distance to at least one other squirmer in this set is less than $2R +R/4$. Finally, we only take the last $2.5\cdot 10^5$ timesteps into account where the system has reached steady state.

Both, the cluster extension (a) and size [inset of (b)], show a pronounced maximum, especially for $N=110$, where clusters are largest. The increase at small torque values leading up to this maximum is quite sharp. As already stated, in this regime clusters start to form due to gyrotaxis and are more short-lived, because the external torque is not strong enough to stabilize them. At and close to the maxima in extension and size, the clusters are rather loosely bound, which is visible in the snapshots for torque value $r_0/R\alpha = 0.32$ in Fig. 2 and in the corresponding videos M1-M3. The clusters strongly vary in size and also in shape with ongoing time. This behavior is reflected by the peak in the normalized standard deviation $\varDelta N_\mathrm {cl}/\langle N_\mathrm {cl}\rangle$ of the mean cluster size as shown in the main plot of Fig. 5(b).

Beyond the maxima of cluster radius and size, both quantities decrease with increasing torque. The clusters become more compact and exhibit less variation also in time, as the decrease in the normalized standard deviation $\varDelta N_\mathrm {cl}/\langle N_\mathrm {cl}\rangle$ shows. This behavior is most clearly visible for $N=110$. For further increasing torque all three quantities then become nearly constant. While the decrease in $\langle N_\mathrm {cl} \rangle$ is not very pronounced, it is stronger for the mean cluster radius, in particular, for $N=110$. Thus the clusters become compactified, while their total number increases, as the last row in Fig. 2 nicely demonstrates. Interestingly, the compactification of the clusters agrees with the location of the dashed line. According to eq. (11) it shows the value of the torque that is just able to balance squirmer rotation due to the stokeslet vorticity of a neighboring touching squirmer. Since squirmers in a cluster also hinder each other from moving, the cluster is stabilized and forms compact objects.

The reason for the decrease of the cluster radius is similar to the two-squirmer case described in Sect. 3.1.1. As the torque increases, squirmers are more vertically oriented and thus their horizontal mobility is further reduced. This has the effect that the motion of squirmers within each cluster is more constrained just as the oscillation amplitude of the squirmer pair also decreases with the torque. In order to follow the vorticity acting on the squirmers, they eventually also evade in the negative *z*-direction and the cluster assumes a stacked arrangement, as we have seen in Fig. 4.

#### Radial distribution function

To investigate squirmer ordering within the clusters and also how the clusters position themselves relative to each other, we calculate the radial distribution function *g*(*r*). Here, *r* is the squirmer distance in a horizontal plane, after projecting all squirmer positions on this plane. Thus, we treat the squirmer system as a two-dimensional system. The two-dimensional radial distribution function for *N* squirmers in an area *A* with positions ${\mathbf {r}}_i$ is defined as [99]

15 $$\begin{aligned} g(r) = \frac{1}{N/A}\langle \sum _{i\ne j} \delta \left( \vert {\mathbf {r}}_i - {\mathbf {r}}_j\vert \right) \rangle , \end{aligned}$$

where $r = | {\mathbf {r}}_i - {\mathbf {r}}_j |$ and the ensemble average is performed over all recorded squirmer configurations in the last $2.5\cdot 10^5$ time steps of the simulations. Note that a constant value of $g(r)=1$ corresponds to the random ordering of an ideal gas.

The radial distribution function gives meaningful results for distances up to ca. *L*/2, thus half the linear box size. Figure 2 shows that the cluster distance $\varDelta$ can assume such values. In order to rule out finite-size effects, we have doubled the linear size of our simulation box and use $L=216a_0$. This increases the cross-sectional area by a factor of 4. Since we simulate 256 squirmers, the area fraction in the horizontal plane is the same as when we use $N=64$ squirmers for our regular box size.

**Fig. 6:**
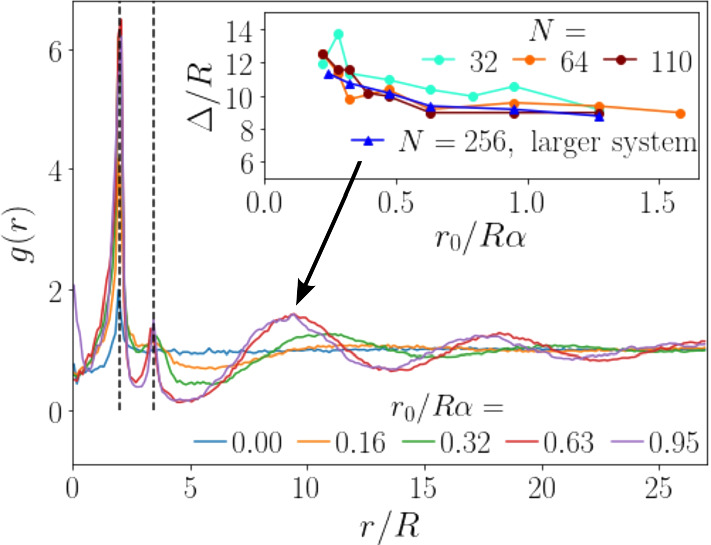
Radial distribution function for several non-dimensional torques for a larger system with 256 squirmers and linear system size $L=216$. Inset: Mean cluster distance $\varDelta$ plotted versus torque for three squirmer numbers at the regular siystem size and $N=256$ at the larger system size. The dashed lines indicate the nearest-neighbor and next-nearest-neighbor distances within a cluster at 2*R* and $2\sqrt{3}R$, respectively

In the main plot of Fig. 6 we show the radial distribution function *g*(*r*) for the larger system for several torque values. In all curves a peak at the nearest neighbor distance $r=2R$ is visible indicated by the dashed vertical line on the left. The blue curve for zero torque then assumes the constant value one at larger distances reflecting the randomly distributed squirmer positions. Already at $r_0/R\alpha =0.16$ a small maximum develops around the next-nearest neighbor distance $2 \sqrt{3}R=3.46 R$ (right dashed line), which becomes more pronounced with increasing torque. In particular, for the two highest torque values, where compact squirmer clusters occur, sharp peaks are visible. Note that both peaks are broadened towards smaller distances due to the partial overlap of the squirmers in the projection plane, when they assume different heights. The overlap is clearly visible in the snapshots of Fig. 2 as already discussed earlier.

Now we discuss the characteristic distances $\varDelta$ between the clusters as revealed by the radial distribution function. Already at $r_0/R\alpha = 0.16$ a weak minimum appears around $r=6R$ followed by a very shallow maximum located between $r=10R$ and $r=15R$. This signals the appearance of squirmer clusters separated from each other. Increasing the torque to $r_0/R\alpha =0.32$ (green curve), this third peak becomes more pronounced and its position shifts to smaller values. Finally, for the two highest torque values ($r_0/R\alpha = 0.63$ and 0.95), where clusters show compact packing, the peaks are identical. They are further shifted to smaller distances and their heights are larger. Interestingly, a further maximum for both torque values is visible at around $r=17R$ and 18*R*, respectively. It corresponds to the next-nearest neighbor shell of the cluster ordering. The maximum cannot be seen in the system with regular size, since all clusters are nearest neighbors due to the periodic boundary conditions.

Now, we focus on the the average cluster-cluster distance $\varDelta$ in the inset of Fig. 6. We plot it versus the dimensionless torque for different squirmer numbers *N* in our regular system size and for the larger system. For $\varDelta$ we use the position of the first maximum in *g*(*r*) connected to cluster ordering. With increasing *N* the curves slightly shift downwards but overall we can state that the cluster distance does not depend on the area density of the squirmers. As we already realized, at small torques, when the clusters start to form, the cluster distance decreases with increasing torque but once compact clusters are formed at $r_0/R\alpha =0.64$ and beyond it stays constant.

The probable explanation for the decrease in $\varDelta$ is that clusters get smaller at larger torques [see Fig. 5 (a)]. Thus, there are more clusters for the same number of squirmers, and the space between them has to shrink. This connects the decrease in cluster distance to the clustering mechanism and to gyrotaxis, which suggests that $\varDelta$ is an instrinsic quantity which emerges from the system parameters. We confirmed this by letting the end configuration for torque $r_0/(R\alpha )=0.28$ evolve further at an increased torque $r_0/(R\alpha )=0.63$. Indeed, the typical cluster distance decreases to the same value as observed for the random initial condition.

#### Influence of squirmer flow fields

**Fig. 7:**
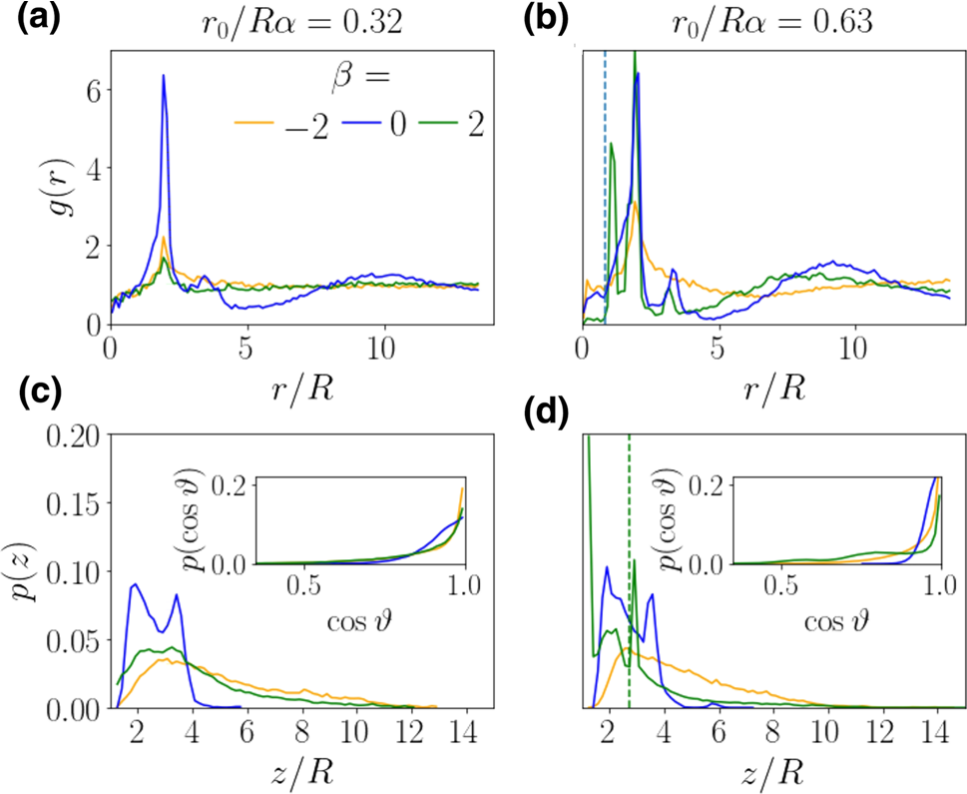
Radial distribution function for $\beta =-2,0,2$ at torque values **a** $r_0/R\alpha = 0.32$ and **b** $r_0/R\alpha = 0.63$. The regular system size with 64 squirmers and $\alpha = 0.8$ is used. Vertical density profiles for the same parameters at **c** $r_0/R\alpha = 0.32$ and d) $r_0/R\alpha = 0.63$. Insets: Distributions of vertical orientations

We consider two squirmer modes in addition to the $B_1$ mode that modify the squirmer type. First, the $B_2$ mode quantified by the parameter $\beta = B_2/B_1$ creates a force dipole in the far field and second, the $C_2$ mode introduces a rotlet dipole in the far field and is tuned via the parameter $\chi = C_2/B_1$, as we explain in Sect. 2.1.1.

**Fig. 8:**
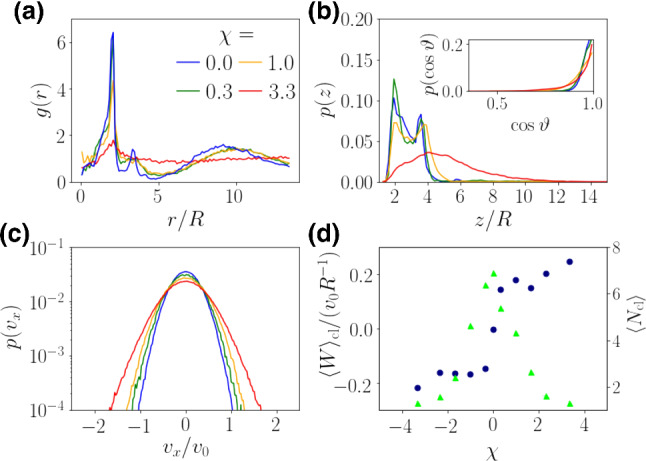
**a** Radial distribution function, **b** vertical density profile, and **c** distribution of horizontal velocities for several values of $\chi$ at $r_0/R\alpha = 0.63$ for the regular system size with 64 squirmers and $\alpha = 0.8$. Inset in (b): distributions of vertical orientations. d) Mean swirling parameter $\langle W\rangle _\mathrm {cl}$ (blue dots), averaged over all clusters and time, and mean cluster size $N_\mathrm {cl}$ (green triangles) plotted versus $\chi$

We again probe the order of the system by calculating the radial distribution functions for pushers, pullers, and rotlet-dipole squirmers. They are shown in Figs. 7 and 8 together with density profiles. We also provide videos of the puller squirmers for $\beta =2$ at torques $r_0/R\alpha =0.32$ (M5) and $r_0/R\alpha =0.63$ (M6) as well as the rotlet-dipole squirmers with $\chi =1.0$ (M7).

$B_2$ **mode** Pusher and puller squirmers generate force-dipole flow fields through which they also interact with each other [65, 67]. In particular, the additional flow vorticity presented in Eq. (8) competes with the external and other hydrodynamic torques and thus contributes to the gyrotactic mechanism. As a consequence, the squirmer type $\beta$ has a profound influence on the radial distribution function *g*(*r*), as Figs. 7 a) and b) demonstrate. At the lower torque value the peak corresponding to the mean cluster distance for neutral squirmers has disappeared completely for $\beta = \pm 2$ and clustering does not exist. The vertical density profiles in Fig. 7 c) underline this: pushers and pullers reach larger heights while neutral squirmers are concentrated close to the botton since their clusters cause collective sinking. In accordance with these findings, video M5 for puller squirmers with $\beta =2$ does not show clustering.

Doubling the external torque [Figs. 7 b) and d)] still does not lead to visible clustering for the pusher system. This agrees with previous findings that cluster formation in pusher systems is generally not favored [40, 60, 100]. However, the behavior of the puller system ($\beta = 2$) changes drastically for the higher torque. First, we now see a weak peak at a distance 8*R* in the radial distribution function in Fig. 7 b) indicating the existence of squirmer clusters. Indeed, we also observe small clusters in video M6. This is in agreement with the density profile in Fig. 7 d), which is now more concentrated close to the bottom wall due to the collective sinking in clusters in contrast to the smaller torque value. The density profile also has two pronounced peaks, one of which is directly at the bottom wall. They indicate the stacked configurations of clusters typically observed for neutral squirmers already at smaller torques. Interestingly, in *g*(*r*) an additional, pronounced peak at a distance below the nearest-neighbor peak at $r=2R$ has developed, which we discuss further below.

The puller clusters have a different structure compared to the neutral squirmer clusters. Video M6 shows pullers in a cluster typically pointing towards the cluster center. This agrees with the typical hydrodynamic puller-puller attraction along the swimmer axis and mutual reorientations towards each other due to the force-dipole vorticity [101]. The tilt of the puller squirmer towards the horizontal is also caused by its near field close to a no-slip wall [102]. The pronounced tilt of the puller axis away from the vertical is also visible in the distribution of vertical orientations in the inset of Fig. 7 d). Even a weak local maximum at $\cos \vartheta < 1$ appears.

Like neutral squirmers, puller clusters develop a stacked configuration. The second peak in the density profile of the pullers in Fig. 7 d) belongs to a second layer of squirmers on top of the bottom layer. Note that the inwards pointing squirmers in the bottom layer induce fluid flow towards the cluster center such that the squirmer from the second layer settles there. Thus, the cluster has a pyramidal three-dimensional structure. Assuming a pyramid with an equiliateral triangle as the base gives a horizontal distance of $\sqrt{3}/2 R$ between the nearest squirmers from the upper and lower layers and a vertical distance of 3/2*R*. We indicate these values as vertical dashed lines in Figs. 7 b) and d), respectively. They agree well with the peaks found from the simulations.

$$C_2$$ **mode**

In Fig. 8 a)-c) we respectively show radial distribution functions, vertical density profiles, and distributions of horizontal velocities for neutral squirmers ($\beta =0$) and several rotlet-dipole parameters $\chi$. Clusters form for all values except $\chi =3.3$, where no peak is visible in the radial distribution function beyond the nearest-neighbor distance. Furthermore, only the vertical distribution of squirmers with $\chi =3.3$ extends to larger heights, whereas it is concentrated close to the bottom for the other $\chi$ values, typical for squirmer clusters. Thus, strong rotlet-dipole flow prevents cluster formation. In addition, Fig. 8 c) shows that with increasing $\chi$ the horizontal velocity components increase, which also contributes to the disappearance of clusters at high $\chi$. This is also indicated in Fig. 8 d), which plots $\langle N_\mathrm {cl}\rangle$ versus $\chi$ (green triangles). The disappearance of the clusters also depends on other parameters. At $r_0/(R\alpha ) = 0.32$ the clusters already disappear at $\chi =1.0$, whereas puller clusters at $\beta =2$ were not observed for the simulated non-zero $\chi$.

For the value $r_0/(R\alpha )=0.63$ depicted in Fig. 8, an inspection of video M7 reveals that already at $\chi =1.0$ squirmers stay more loosely bound to each other and clusters are less compact. Furthermore, we observe that squirmers in a cluster swirl around the cluster center in anti-clockwise direction. In Eq. (14) we show the angular velocity, which a rotlet dipole close to a no-slip wall experiences due to the image flow. It is known to induce circular trajectories [66], for example, of *E.coli* bacteria [73, 103]. While *E.coli* swim in circles with their orientation vector parallel to the wall, our squirmers have a strong vertical bias. According to Eq. (14) this corresponds to an angular velocity with reversed sign, as long as $\cos \vartheta$ exceeds $\sqrt{1/3}\approx 0.58$. This is indeed the case, as the inset in Fig. 8 (b) shows. The constant in-plane reorientation combined with the squirmers’ self-propulsion results in circular swimming trajectories and in our simulations creates the swirling clusters.

We introduce the swirling parameter *W* for a given cluster with a cluster center at $\overline{{\mathbf {r}}}$ by averaging the angular velocity of the circling squirmers in the cluster:

16 $$\begin{aligned} W = \left\langle \frac{ \left( {\mathbf {r}} - \mathbf {\overline{r}}\right) \times {\mathbf {v}}}{\vert {\mathbf {r}} - \mathbf {\overline{r}} \vert ^2} \cdot {\mathbf {e}}_z\right\rangle \, . \end{aligned}$$

We only take clusters with a cluster size $N_\mathrm {cl} > 2$ into account. In Fig. 8 d) we show this quantity averaged over all clusters and $2.5\cdot 10^5$ timesteps, normalized by the inverse ballistic time scale $v_0/R$. Clearly, at $\chi =0$, the squirmers do not swirl. For non-zero $\chi$ the normalized swirling parameter jumps to a value with magnitude around $0.15 v_0/R$, and afterwards fluctuates in a narrow region between $0.15 v_0/R$ and $0.2v_0/R$. For $|\chi |=3.3$, squirmers group together for short times, even though no long-term stable clusters emerge. Therefore we can still measure a value of *W*.

Since the chirality of the squirmer system changes with the sign of $\chi$, we can induce clock-wise motion with $\chi < 0$, corresponding to $\langle W\rangle <0$. We show this case in video M8, where $\chi =-1$.

#### Influence of gravity

**Fig. 9:**
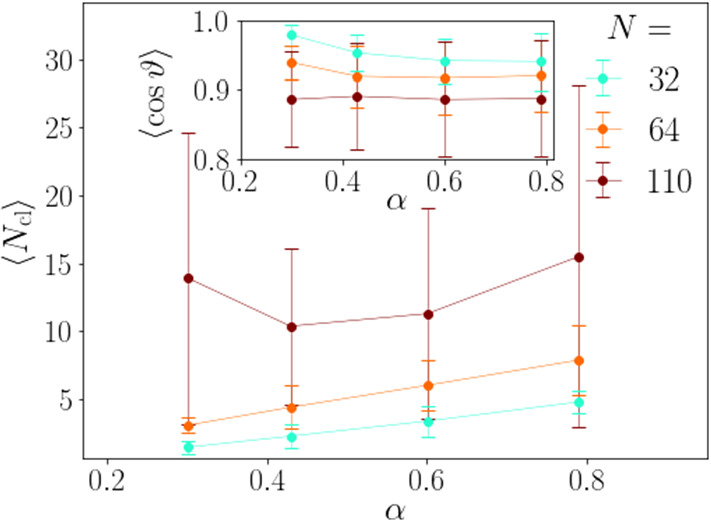
Mean cluster size of neutral squirmers plotted versus $\alpha$ for several values of total squirmer number *N* at $r_0/R\alpha =0.32$. Inset: Mean vertical orientation of squirmers plotted versus $\alpha$

The parameter $\alpha = v_0 / v_\mathrm {sed}$ determines how strongly squirmers are confined to the bottom wall. On the one hand, gravity induces the stokeslet flow via which squirmers also interact with each other hydrodynamically. Thus, at smaller $\alpha$ one expects squirmers to be more tilted against the vertical due to the stronger flow vorticity. On the other hand, at stronger gravity squirmers are closer to the bottom wall and therefore the hydrodynamic interaction with the no-slip wall also forces a stronger upright orientation in addition to the gravitational torque, see Eq. (13) [21, 67]. Thus, the behavior of the system is determined by these two effects.

In Fig. 9 we plot the mean cluster size $\langle N_\mathrm {cl} \rangle$ versus $\alpha$. It increases monotonically for $N=32$ and $N=64$. Thus, clusters become smaller at higher gravity and finally disintegrate into single squirmers. In order to find the reason for this behavior, we measured the orientational distributions and show the mean vertical orientation in the inset. The general trend is that for decreasing $\alpha$ (increasing gravity), the squirmer orientation is more aligned with the vertical. Thus, the stronger vertical alignment of neutral squirmers due to their hydrodynamic interactions with the wall is able to counterbalance the action of the stronger stokeslet vorticities. This results in more compact and smaller clusters, similar to the effect of larger gravitational torques in Sect. 3.2.1.

For the system with $N=110$ squirmers the mean cluster size seems to increase again for the lowest value $\alpha =0.3$. However, due to the large error bars this increase is not secured. A possible reason could be that due to the stronger confinement to the bottom wall and the larger areal density, the squirmers are in close contact to each other which increases the measured cluster size.

We note that as the clusters become smaller, they also start to rotate as a whole. In particular, we observe rotating squirmer trimers at large gravity. We mention them since they resemble the spinner states found at very high gravity in lattice-Boltzmann simulations [104]. In these studies the vertical alignment of neutral squirmes is solely due to their hydrodynamic interactions with the wall [see Eq. (13)]. Furthermore, in the limit of very large gravity ($\alpha \rightarrow 0$), our squirmer system reaches the regime of Ref. [47], where at zero external torque a quasi-hexagonal monolayer (Wigner fluid) of squirmers with upright orientation is formed.

## Conclusions

We have investigated the cluster formation of bottom-heavy squirmers under strong gravity floating above the bottom surface. Already a squirmer pair mimicking the minuet dance, found in experiments with the Volvox algae, reveals the basic gyrotactic mechanism [54, 105]. While the stokeslet vorticity from the squirmer neighbor tilts the squirmer towards the neighbor so that they swim towards each other, the gravitational torque stabilizes the upright orientation. This enables horizontal oscillations of the squirmer positions.

At higher squirmer numbers, the vorticity-induced mutual attraction leads to the formation of squirmer clusters, however only for sufficiently large gravitational torques. We quantify the cluster properties by the two-dimensional density distribution, the mean cluster size, and the radial distribution function, from which we extract the mean cluster distance. Our simulations find that horizontal motion within the clusters decreases with increasing torque and the clusters become more compact. The cluster size has a maximum at a finite torque that is close but below the value where the vorticity-induced rotation of touching neighbors can be balanced. The maximum standard deviation of the cluster size at the same torque value shows the volatility of the clusters in the emerging cluster state. Increasing the torque further, squirmers in the clusters are less mobile. The clusters become more compact and ultimately the cluster size reaches a constant value for each density. The mean distance between the clusters also decreases with increasing torque and saturates. Interestingly, the areal density has little effect on the mean cluster distance.

Furthermore, we investigated how force and rotlet dipoles influence cluster formation. Puller squirmers require larger torques in order to form clusters, because their additional flow-field vorticity adds to the effective attraction and thus the stabilizing gravitational torque has to be larger. Their clusters are situated closer to the bottom wall where hydrodynamic wall interactions tilt squirmers towards each other. In contrast, pushers do not form noticeable clusters in our simulations. Squirmers with a rotlet dipole also form clusters for small dipolar strengths and due to their interaction with the no-slip wall perform swirling motion. Finally, for increasing gravitational strength squirmers move closer to the bottom wall. The hydrodynamic wall interactions contribute to the vertical alignment of neutral squirmers, which also decreases the cluster size. In conclusion, the swimmer hydrodynamics has a profound effect on the cluster formation when the occurring hydrodynamic multipoles besides the stokeslet also contribute to the flow vorticity, which has to be balanced by the gravitational torque.

Gyrotactic cluster formation could be realized for microswimmers, such as Janus particles or Volvox algae, which are indeed bottom-heavy [45, 54]. Janus particles also offer the possibility to tune the strength of the gravitational torque, while for Volvox algae it exhibits a natural variation. For an L-shaped particle the torque was controlled by changing the relative arm lengths and thus the effective lever arm [33]. However, the realization of the external torque is not decisive for our results. For example, phototaxis is an alternative way of inducing orientational alignment and the strength of the vertical reorientation can be controlled by light intensity [106, 107]. But here, the size and orientation of the part of the swimmer, which is illuminated, also influences the torque, as was shown for a phototactic Janus particle [108]. Microswimmers casting shadows on each other, influence their collective dynamics. Furthermore, gradients in oxygen concentration also orient bacteria along the vertical, which was observed for *B. subtilis* swimming upwards towards an air-liquid interface at the top [59]. However, in general, any chemotaxis towards chemical fields, such as nutrients, can profoundly affect the dynamics of microswimmers as exemplified by the effect of trail avoidance in active emulsion droplets [109]. These droplets also form floating, spontaneously rotating clusters, when the concentration of the surfactant fuel is high enough [97]. Thus, while we have identified the generic hydrodynamic mechanism of gyrotactic cluster formation, the influence of chemical fields needs further study.

Finally, we mention two possible extensions of our investigations. Biological microswimmers typically move in non-Newtonian fluids [110, 111]. Therefore, it is worthwhile to investigate, how robust gyrotactic cluster formation is for squirmers in complex fluids. This requires to extent the method of MPCD to viscoelastic media [80]. Furthermore, current research also explores the dynamics of non-spherical swimmers [112–114], including squirmer rods [86, 115]. The collective motion of rod-shaped particles is strongly influenced by their steric interactions. Under a gravitational torque, this suggests that the vertical orientation is even more stable in a dense suspension with smectic order, because collisions with neighboring rods counteract tilting against the vertical. Therefore, the interesting question arises how this affects the formation of gyrotactic clusters.

## Supplementary Information

Below is the link to the electronic supplementary material.

Supplementary file 1 (mp4 3916 KB)

Supplementary file 2 (mp4 11219 KB)

Supplementary file 3 (mp4 17318 KB)

Supplementary file 4 (mp4 19740 KB)

Supplementary file 5 (mp4 17723 KB)

Supplementary file 6 (mp4 12591 KB)

Supplementary file 7 (mp4 15895 KB)

Supplementary file 8 (mp4 16118 KB)

## Declarations

### Data Availability Statement

This manuscript has data included as electronic supplementary material. The online version of this article contains supplementary material, which is available to authorized users.
