# Correction to: Gyrotactic cluster formation of bottom-heavy squirmers

**DOI:** 10.1140/epje/s10189-022-00210-5

**Published:** 2022-07-08

**Authors:** Felix Rühle, Arne W. Zantop, Holger Stark

**Affiliations:** Institut für Theoretische Physik, Technische Universität Berlin, Hardenbergstr. 36, 10623 Berlin, Germany

## Correction to:The European Physical Journal E (2022) 45:1-14 10.1140/epje/s10189-022-00183-5

Equation (9) should read the balance between the angular velocities from the external bottom-heavy torque (Eq. (5)) and from the Stokeslet vorticity (Eq. (6)). However, a factor of $\frac{3}{4}$ was erroneously omitted. The corrected equation reads

9 $$ \frac{3}{4}\frac{{v_{0} }}{R}\frac{{r_{0} }}{R\alpha }\sin \vartheta = \frac{3}{4}\frac{{v_{0} }}{\alpha }\frac{R}{{r^{2} }}. $$

Consequently, the two subsequent equations, where *r *= 2*R*, should read

10 $$ \sin \vartheta = \frac{{1{/(4}\alpha {)}}}{{r_{0} {/(}R\alpha {)}}}. $$

and

11 $$r_{0} {/(}R\alpha {)} \ge {(4}\alpha {)}^{ - 1} .$$

The corrected balance of angular velocities requires Fig. 5 to be updated. We have plotted black dotted vertical lines for the corrected value of the equality condition from Eq. (11) and show the incorrect line from the original manuscript in red.

**Fig. 5:**
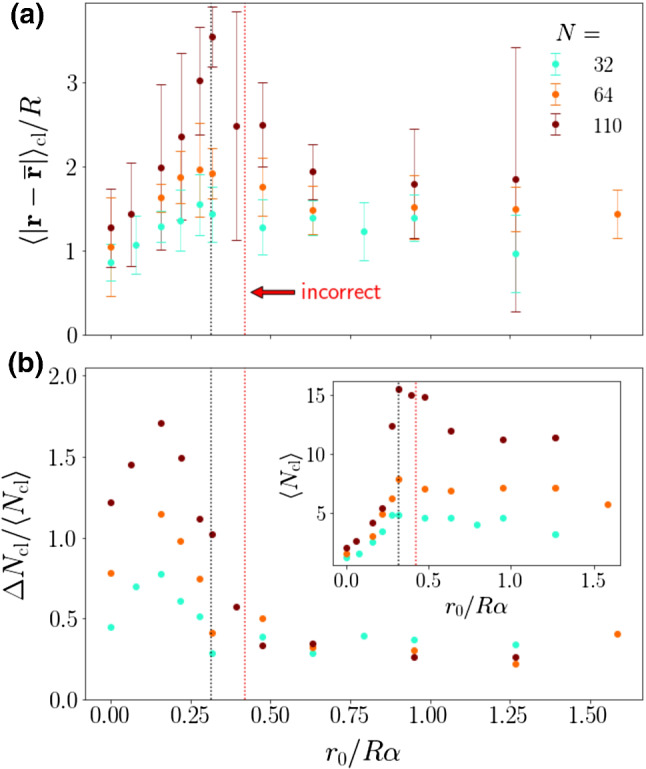
**a** Mean cluster radius $\langle \vert {\mathbf {r}}-{\mathbf {\overline{r}}}\vert \rangle _{\mathrm {cl}}$ in units of *R* plotted versus torque value $r_0/R\alpha$ for different squirmer numbers *N*. **b** Normalized standard deviation $\varDelta N_{\mathrm {cl}}/\langle N_{\mathrm {cl}} \rangle$ and inset: mean number of squirmers in a cluster $\langle N_{\mathrm {cl}} \rangle$. The dotted vertical lines show the equality condition of Eq. (11) for $\alpha =0.8$. The red dotted line shows the erroneous condition

## Conclusion

The angular velocity balance between Stokeslet vorticity and bottom-heaviness results in a lower bound for the rescaled torque $r_{0} {/(}R\alpha {)}$ that is lower than originally presented by a factor of $\frac{3}{4}$. The conclusions of the original paper are unaffected.

